# Pharmacological Targeting of the NMDAR/TRPM4 Death Signaling Complex with a TwinF Interface Inhibitor Prevents Excitotoxicity-Associated Dendritic Blebbing and Organelle Damage

**DOI:** 10.3390/cells14030195

**Published:** 2025-01-28

**Authors:** Omar A. Ramírez, Andrea Hellwig, Zihong Zhang, Hilmar Bading

**Affiliations:** 1 Department of Neurobiology, Interdisciplinary Center for Neurosciences (IZN), Heidelberg University, 69120 Heidelberg, Germany; ramirez@nbio.uni-heidelberg.de (O.A.R.); hellwig@nbio.uni-heidelberg.de (A.H.); zhang@nbio.uni-heidelberg.de (Z.Z.); 2 Network Aging Research, Heidelberg University, 69115 Heidelberg, Germany

**Keywords:** glutamate neurotoxicity, TwinF interface inhibitor, NMDAR/TRPM4 complex, organelle damage, mitochondria, endoplasmic reticulum, ER–mitochondria contact sites

## Abstract

Focal swellings of dendrites (“dendritic blebbing”) together with structural damage of mitochondria and the endoplasmic reticulum (ER) are morphological hallmarks of glutamate neurotoxicity, also known as excitotoxicity. These pathological alterations are generally thought to be caused by the so-called “overactivation” of N-methyl-D-aspartate receptors (NMDARs). Here, we demonstrate that the activation of extrasynaptic NMDARs, specifically when forming a protein–protein complex with TRPM4, drives these pathological traits. In contrast, strong activation of synaptic NMDARs fails to induce cell damage despite evoking plateau-type calcium signals that are comparable to those generated by activation of the NMDAR/TRPM4 complex, indicating that high intracellular calcium levels per se are not toxic to neurons. Using confocal laser scanning microscopy and transmission electron microscopy, we show that disrupting the NMDAR/TRPM4 complex using the recently discovered small-molecule TwinF interface inhibitor FP802 inhibits the NMDA-induced neurotoxicity-associated dendritic blebbing and structural damage to mitochondria and the ER. It also prevents, at least in part, the disruption of ER–mitochondria contact sites. These findings establish the NMDAR/TRPM4 complex as the trigger for the structural damage of dendrites and intracellular organelles associated with excitotoxicity. They also suggest that activation of the NMDAR/TRPM4 complex, in addition to inducing high-amplitude, plateau-type calcium signals, generates a second signal required for glutamate neurotoxicity (“two-hit hypothesis”). As structural damage to organelles, particularly mitochondria, is a common feature of many human neurodegenerative diseases, including Alzheimer’s disease and amyotrophic lateral sclerosis (ALS), TwinF interface inhibitors have the potential to provide neuroprotection across a broad spectrum of these diseases.

## 1. Introduction

**Figure 1:**
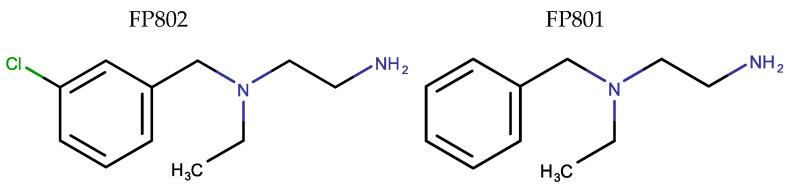
Structural formulas of the compounds used in this study. Small molecule TwinF interface inhibitor FP802 (**left**) and inactive compound FP801 (**right**).

Glutamate neurotoxicity, also known as excitotoxicity, is linked to NMDAR activity and is often attributed to “overactivation” of these receptors [1]. The term “overactivation” is poorly defined and, in the literature, is often used in the context of conditions where cultured neurons are stimulated through the bath application of glutamate or N-methyl-D-aspartate (NMDA). These stimulation protocols are believed to cause a “calcium overload” in neurons, which acts as the trigger of cell death processes. However, NMDAR-induced prolonged, high-amplitude, plateau-type calcium signals, very similar to those typically observed with toxicity-inducing bath application of glutamate or NMDA, can also be generated by high-frequency bursts of action potential firing. Notably, these burst-induced very strong global calcium signals do not initiate cell death signaling [2]. Thus, it is neither the “overactivation” of the NMDAR nor the sustained high-amplitude calcium signals per se that trigger neurotoxicity. Instead, the reason is that bath application of glutamate or NMDA activates NMDARs located outside synaptic contacts: the extrasynaptic NMDARs [2]. Action potential firing inducing strong synaptic activity does not activate extrasynaptic NMDARs under physiological conditions with intact glutamate re-uptake systems. This explains why even intense activation of synaptic NMDARs does not lead to cell death, but instead promotes neuronal survival through induction of an acquired neuroprotection gene program [3,4,5,6]. The reason why extrasynaptic NMDARs induce cell death pathways is their interaction with TRPM4 [7,8]. Disruption of the NMDAR/TRPM4 complex using a recently discovered new pharmacological class of small molecules named TwinF interface inhibitors eliminates the toxic signaling of extrasynaptic NMDARs [7]. In this study we used the TwinF interface inhibitor FP802 and the inactive control compound FP801 (Figure 1) [7,8,9] to investigate the contribution of the NMDAR/TRPM4 complex to the structural pathology associated with glutamate/NMDA neurotoxicity. In particular, we used electron microscopy and confocal live-cell imaging to monitor dendritic blebbing, structural damage of mitochondria and the endoplasmic reticulum (ER), and the loss of mitochondria–ER contact sites.

## 2. Materials and Methods

### 2.1. Cell Culture

Primary rat hippocampal cultures were prepared from newborn animals, as previously described [10]. Briefly, hippocampal neurons were seeded on poly-D-lysine/laminin-coated coverslips and maintained in Neurobasal-A medium (Thermo Fisher Scientific, New York, NY, USA; 10888) supplemented with B27 (Thermo Fisher Scientific, New York, NY, USA; 12587-010), 0.5 mM glutamine (Sigma-Aldrich, Taufkirchen, Germany; G7513), 1% rat serum (Biowest, Nuaillé, France; S2150), and 0.5% penicillin/streptomycin (Sigma-Aldrich, Taufkirchen, Germany; P0781) in a humified 5% CO_2_ atmosphere at 37 °C. On day in vitro (DIV) 3, cytosine β-D-arabinofuranoside (AraC) (Sigma-Aldrich, Taufkirchen, Germany; C1768) was added to prevent glial cell proliferation. On DIV 8, the medium was changed to transfection medium (TM) containing 88% buffered salt/glucose/glycine solution [mM: 10 HEPES, pH 7.4, 114 NaCl, 26.1 NaHCO_3_, 5.3 KCl, 1 MgCl_2_, 2 CaCl_2_, 30 glucose, 1 glycine, 0.5 sodium pyruvate, and 0.001% phenol red], 10% phosphate-free Eagle’s minimum essential medium (Sigma-Aldrich, Taufkirchen, Germany; M4655), 1.5% ITS [insulin (75 ng/mL), transferrin (75 ng/mL), sodium selenite (75 pg/mL)] (Sigma-Aldrich, Taufkirchen, Germany; I3146), and 0.5% penicillin/streptomycin.

### 2.2. Transfection of Primary Hippocampal Neurons

Primary rat hippocampal neurons were transfected on DIV 10 with an EGFP-encoding plasmid using Lipofectamine 2000 (Thermo Fisher Scientific, New York, NY, USA; #11668019). DNA–lipid complexes were prepared by combining 1 µg EGFP plasmid with Lipofectamine in culture medium, followed by incubation for 20–30 min at room temperature. Neurons were exposed to the transfection mixture for 20 min at 37 °C, then washed and maintained in fresh medium. EGFP expression was evaluated 24 h post-transfection to confirm efficiency.

### 2.3. Recombinant Adeno-Associated Viruses (rAAVs) and Reagents

Viral particles were produced and purified as described previously [11]. Primary neurons plated on coverslips (diameter 12 mm) in 4-well plates were infected on DIV 8 with the following recombinant adeno-associated viruses alone or in combinations: rAAV-hSyn-mCherry-Mito-7 (modified from Addgene; #55102), rAAV-hSyn-ER-mNeonGreen (modified from Addgene; #137804), rAAV-hSyn-SPLICSS-P2A-ER-mito [12], N-methyl-D-aspartic acid, NMDA (Hello Bio, Dunshaughlin, Republic of Ireland; HB0454), 4-aminopyridine, 4-AP (Sigma-Aldrich, Taufkirchen, Germany; A0152), bicuculline (Hycultec, Beutelsbach, Germany; HY-N0219), FP801 (CCN(CCN)CC1=CC=CC=C1), and FP802 (ClC1=CC(CN(CC)CCN)=CC=C1) (FundaMental Pharma, Heidelberg, Germany).

### 2.4. Confocal Live-Cell Imaging

Cultured hippocampal neurons were imaged using a TCS SP8 confocal laser scanning microscope coupled to a DM6 CFS Flex upright stand controlled by LAS X software version 3.5.7.23225 (all from Leica Microsystems, Wetzlar, Germany). Live imaging experiments were performed using an HC APO L 63×/0.9 water immersion objective or HC FLUOTAR L 25×/0.95 water immersion objective (Leica Microsystems, Wetzlar, Germany). Briefly, coverslips with DIV 11-13 hippocampal neurons were transferred into an OAC-1 laminar perfusion chamber (Science Products GmbH, Hofheim, Germany) with the passive perfusion input adjusted to 3 mL/min and with the output connected to a vacuum pump (Merck KGaA, Darmstadt, Germany; XF5423050). The chamber was filled with the CO_2_-independent salt/glucose/glycine imaging medium [mM: 140.1 NaCl, 5.3 KCl, 1 MgCl_2_, 2 CaCl_2_, 10 HEPES, 1 glycine, 30 glucose, and 0.5 sodium pyruvate] and mounted on the microscope stage at 20–22 °C. GFP, ER-mNeonGreen, and SPLICSS-P2A-ER-mito were excited by a 488 nm laser at 4–8% of the maximum intensity, and emitted light was detected at 504–524 nm. MCherry-Mito-7 was excited by a 594 nm laser at 10–15% of the maximum intensity, and emission was detected at 600–620 nm. For SPLICSS-P2A-ER-mito and mCherry-Mito-7 double-transduced neurons, scans were made simultaneously using 2 detectors. All images were acquired using 1024 × 125 pixels, with square pixels of dimensions x, y = 34 nm. The mitochondria and ER imaging protocols were as follows: One positive labeled neuron was chosen per field of view, and one frame spanning the initial segment of a primary dendritic branch was collected every 2 s over a time period of 10 min. A 2 min baseline was acquired, followed by 2 min drug application and 6 min washing. Treatments with 30 μM NMDA in the presence or absence of 10 μM FP801 or FP802 were applied. Images were saved as 8-bit TIFF files and deconvolved using Huygens Professional software (SVI, Hilversum, Netherlands) for data analysis. Primary dendritic segments were outlined, their mitochondria and ER content were segmented, and their shapes analyzed with the open-source software ImageJ. The experimenter was blinded until all analyses were completed. Statistical significance was assessed by repeated-measures t-test. Dendritic blebbing experiments were performed in EGFP-transfected rat neurons. Z-stacks of 3 images spaced by 2 µm were taken every 10 s during 2 min baseline recordings (in imaging medium in the presence or absence of FP802) followed by a 10 min treatment with 30 µM NMDA in the presence or absence of 30 µM FP802. The resulting z-stacks were projected into single planes using the maximum intensity before undergoing visual inspection for the presence of blebs.

### 2.5. Electron Microscopy

DIV 12-13 neurons grown on plastic dishes were washed once with imaging medium and treated with 30 µM NMDA in the presence or absence of 10 µM FP801 or FP802 for 2 min. As control conditions, neurons were either left untreated or treated with 50 µM bicuculline plus 500 µM 4-AP for 2 min. Neurons were fixed with 2% glutaraldehyde in 0.1 M sodium phosphate buffer, pH 7.4, and washed with 0.1 M sodium phosphate buffer. Neurons were post-fixed with 1% OsO_4_, 1.5% K_4_Fe(CN)_6_, contrasted with uranyl acetate, dehydrated with a graded dilution series of ethanol, and embedded into glycid ether 100-based resin. Ultrathin sections were cut with a Reichert Ultracut S ultramicrotome (Leica Microsystems, Wetzlar, Germany) and contrasted with uranyl acetate and lead citrate. Specimens were visualized using an electron microscope, EM 10 CR (Zeiss, Jena, Germany), equipped with a 1K CCD camera (Tröndle Restlichtverstärkersysteme, Moorenweis, Germany) at an acceleration voltage of 60 KV. The software ImageSP version 1.0.30.31 (SYSPROG) was used. Electron microscopy images were analyzed with FIJI version 1.54f using the CLAHE contrast enhance and the brush tool to manually define the areas of the ER and mitochondria. Only mitochondria and ER that were located within longitudinally sectioned dendrites or in somata were included in the analysis. Two different shape descriptors were used: (1) aspect ratio (AR), i.e., the aspect ratio of the particle’s fitted ellipse, calculated by [Major Axis]/[Minor Axis]; and (2) circularity, i.e., 4π × [Area]/[Perimeter]^2^, with a value of 1.0 indicating a perfect circle, where, as the value approaches 0.0, it indicates an increasingly elongated shape. The experimenter was blinded until all analyses were completed. Statistical analysis was performed using the Kruskal–Wallis test with multiple comparisons.

## 3. Results

### 3.1. TwinF Interface Inhibitor FP802 Inhibits NMDA-Induced Dendritic Blebbing

**Figure 2:**
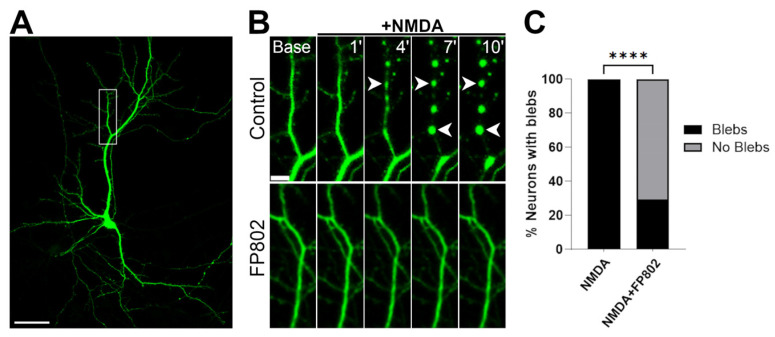
The TwinF interface inhibitor FP802 prevents dendritic blebbing induced by NMDA. (**A**) Representative EGFP-transfected DIV 13 hippocampal neuron. Scale bar, 50 μm. (**B**) Dendrite indicated by a rectangle in (**A**) at the beginning of a 2 min baseline (Base, upper left panel) and at 1, 4, 7, and 10 min after stimulation with NMDA (30 µM) without (upper panels) or with 30 μM FP802 treatment (lower panels). Blebs (examples are indicated with arrowheads) were evident at 4, 7, and 10 min in the control condition, but were absent in the presence of FP802. Scale bar, 10 μm. (**C**) Quantification of dendritic blebs after 10 min of NMDA treatment. Data are from three independent biological replicates, each with 6–8 coverslips per condition (N = 22 for NMDA and N = 24 for NMDA + FP802). The data were analyzed using the Kolmogorov–Smirnov test (**** *p* < 0.0001).

EGFP-transfected rat hippocampal neurons (DIV 13) (Figure 2A) were pretreated with or without FP802 for 5 min before exposure, via constant perfusion for 10 min, to 30 µM NMDA in the absence or presence of the TwinF interface inhibitor FP802 (30 μM) [7,8,9]. Under these conditions, dendritic blebbing started after about 4 min of NMDA treatment, and persisted until the end of the recording (Figure 2B, upper panels). FP802 treatment prevented NMDA-induced dendritic blebbing (Figure 2B, lower panels, and 2C).

### 3.2. TwinF Interface Inhibitor Protects from NMDA-Induced Organelle Damage

**Figure 3:**
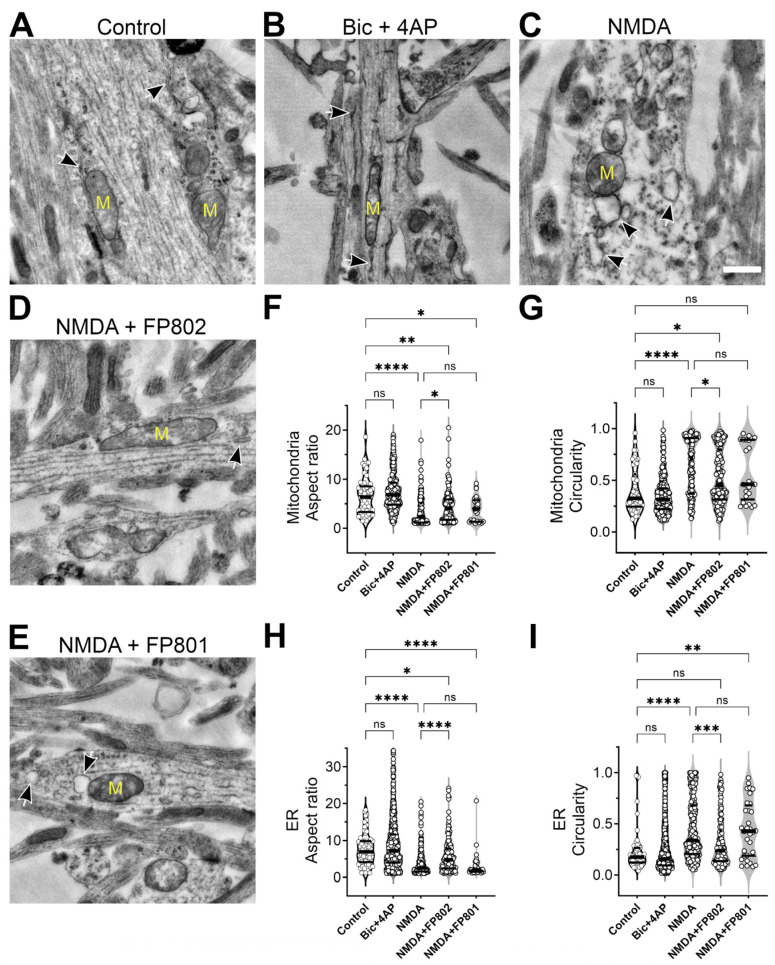
FP802 inhibits NMDA-induced structural damage of organelles (mitochondria and ER) in dendrites. Primary rat hippocampal neurons were incubated in imaging medium alone (**A**–**C**) or medium containing 10 µM FP802 (**D**) or FP801 (**E**) for 1 min and then either left untreated (**A**) or treated with 30 µM NMDA (**C**–**E**) or 50 µM bicuculline plus 500 µM 4AP (Bic + 4AP) (**B**) for 2 min and then immediately fixed for transmission electron microscopy imaging. Scale bar represents 500 nm. Mitochondria (yellow “M” on (**A**–**E**)) and endoplasmic reticulum (black/white arrows on (**A**–**E**)) areas are outlined and aspect ratio (**F**,**H**) and circularity (**G**,**I**) are measured and plotted. Each plot shows the median (thick horizontal line) and quartiles (thin horizontal lines). ****, ***, **, *, ns represent <0.0001, <0.001, <0.01, <0.05, not significant, respectively.

We used electron microscopy (EM) to assess at the ultrastructural level possible damage to dendritic mitochondria and the ER in hippocampal neurons exposed for 2 min to 30 µM NMDA. We observed swellings of both mitochondria and the ER that were virtually absent in untreated control neurons (Figure 3A,C). Morphometric analysis of the aspect ratio or “elongation” and the circularity or “roundness” of mitochondria and the ER revealed significant shape differences consistent with organellar swelling (Figure 3F–I). We found that, after NMDA stimulation, the mitochondria aspect ratio decreased and circularity increased. Similarly, the ER transited from fine and elongated tubular structures to swollen blobs with irregular structures (arrows on Figure 3A,C). To determine whether the prolonged, high-amplitude, plateau-type calcium signals induced by bath application of NMDA and the subsequent activation of extrasynaptic NMDARs are responsible for organelle damage, we employed an alternative stimulation paradigm that generates comparable calcium signals without engaging extrasynaptic NMDARs. Neurons were treated with a combination of the GABA_A_ receptor blocker, bicuculline (Bic), and 4-aminopyridine (4AP), a weak blocker of voltage-activated K^+^ channels. This treatment leads to high-frequency bursts of action potential firing and stimulation of synaptic, but not extrasynaptic, NMDARs, resulting in calcium increases very similar to those obtained with bath application of NMDA or glutamate [2]. We found that Bic/4AP, which strongly promotes neuronal survival through activation of a neuroprotective gene program [13], does not affect organelle structures (Figure 3B,F–I), suggesting that it is not the sustained high calcium signal per se, but the stimulation of extrasynaptic NMDARs that is responsible for the damage. To investigate this further, we pretreated neurons with FP802, which detoxifies extrasynaptic NMDARs by disrupting their complex formation with TRPM4 [7,9]. We found that FP802, but not the inactive control compound FP801, prevented the deleterious effects of NMDA bath application on mitochondria and the ER (Figure 3D–I). These results strongly suggest that stimulation of extrasynaptic NMDARs can induce organelle damage. They also indicate that, in addition to a high calcium signal, whose contribution to excitotoxicity is well documented [14], stimulation of extrasynaptic NMDARs in complex with TRPM4 may generate a second signal that, together with high calcium, initiates mitochondrial and ER damage (the “two-hit hypothesis”).

**Figure 4:**
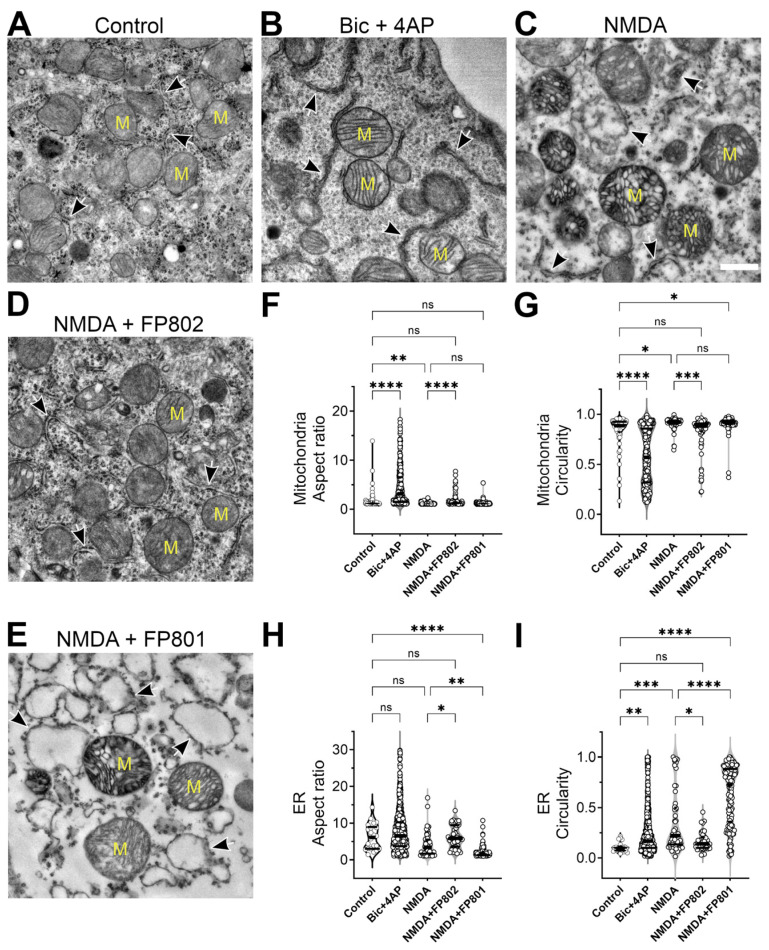
FP802 inhibits NMDA-induced structural damage of organelles (mitochondria and ER) in the cell soma. Primary rat hippocampal neurons were incubated in imaging medium (**A–C**) or medium containing 10 µM FP802 (**D**) or FP801 (**E**) for 1 min and either left untreated (**A**) or treated with 30 µM NMDA (**C**–**E**) or 50 µM bicuculline plus 500 µM 4AP (Bic + 4AP) for 2 min and then immediately fixed for transmission electron microscopy imaging. Scale bar, 500 nm. Mitochondria (yellow “M” on (**A**–**E**)) and endoplasmic reticulum (black/white arrows on (**A**–**E**)) areas are outlined and aspect ratio (**F**,**H**) and circularity (**G**,**I**) are measured and plotted. Each plot shows the median (thick horizontal line) and quartiles (thin horizontal lines). ****, ***, **, *, ns represent <0.0001, <0.001, <0.01, <0.05, not significant, respectively.

Analysis of organelles located in the somatic region of the neurons also revealed NMDA-induced ultrastructural changes. Similar to the changes in the dendrites, somatic mitochondria showed decreased aspect ratios and increased circularity upon NMDA challenge (Figure 4A,C,F,G). The somatic ER also underwent an increase in circularity and showed a trend towards a decreased aspect ratio, which, however, was not statistically significant (Figure 4A,C,H,I). All NMDA-induced structural alterations were inhibited by FP802 and not FP801, further underscoring the importance of the extrasynaptic NMDAR/TRPM4 complex in structural damage (Figure 4D–I). It is worth noting that, after Bic/4AP treatment, mitochondria in the somatic region became more elongated (increased aspect ratios) and showed decreased circularity (Figure 4B,F,G), whereas somatic ER increased circularity and appeared more elongated, although the changes in aspect ratios were not statistically significant (Figure 4B,H,I). Thus, the pro-survival stimulation paradigm Bic/4AP [13] induces structural changes, particularly in somatic mitochondria, that are opposite to those induced by NMDA toxicity.

**Figure 5:**
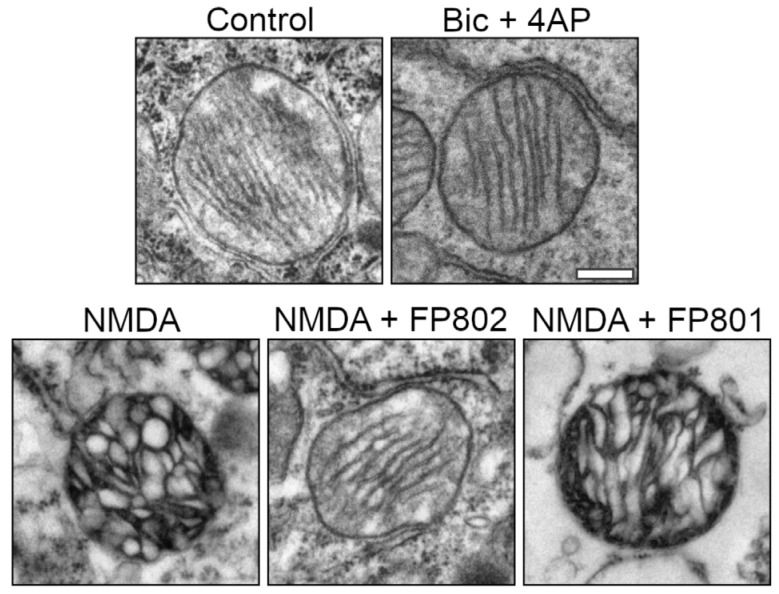
TwinF inhibitor FP802 reduces NMDA-induced disorganization and swelling of mitochondrial cristae. Representative micrographs of somatic mitochondria showing the cristae ultrastructure of control neurons, neurons treated for 2 min with 50 µM bicuculline plus 500 µM 4AP (Bic + 4AP), and neurons treated for 2 min with NMDA in the absence and presence of FP802 or FP801. Scale bar, 250 nm.

We also examined the intramitochondrial structure, focusing on the cristae. In control neurons and in neurons after Bic/4AP treatment, mitochondria displayed a virtually regular arrangement of thin, parallel-oriented cristae (Figure 5, upper row). In contrast, 2 min after NMDA treatment, the mitochondrial cristae became swollen and heterogeneously distributed. These dramatic alterations could be prevented by FP802 but not FP801 (Figure 5, lower row).

### 3.3. Live Imaging of NMDA-Induced Structural Changes of Mitochondria and ER

**Figure 6:**
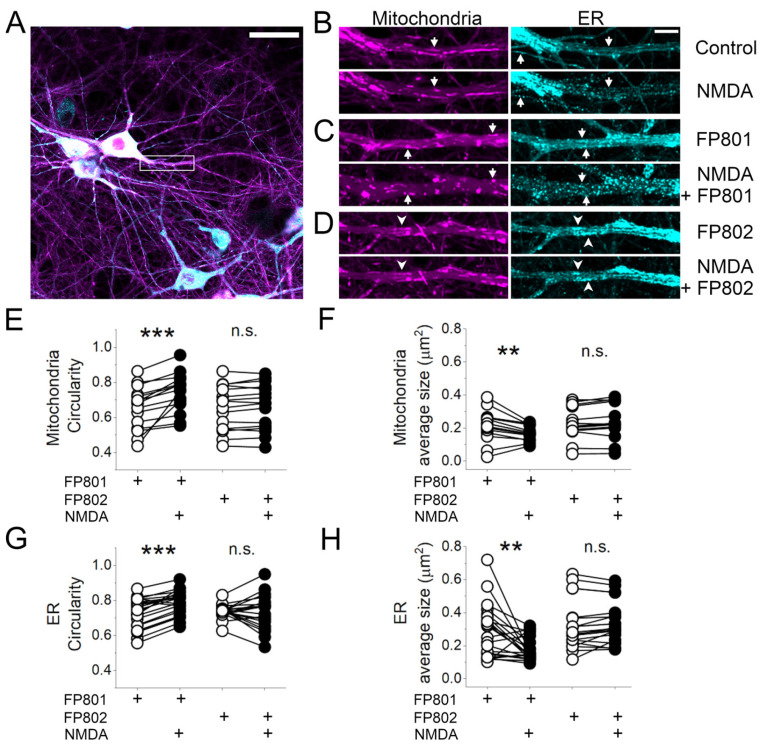
FP802 inhibits the breakdown of dendritic organelle integrity following NMDA treatment. (**A**) Primary rat hippocampal neurons were infected on DIV 8 with rAAVs carrying the sequences encoding for a mitochondrial marker (mito-mCherry), shown in magenta, and an endoplasmic reticulum marker (ER-mNeonGreen), shown in cyan. On DIV 13, neurons were transferred to an imaging chamber with constant perfusion and a time series was acquired every 2 s with a confocal microscope. A representative image shows a primary dendrite (rectangle). Scale bar represents 30 µm. (**B**) Comparison of the dendrite indicated in (**A**) before (upper panels) and after 2.5 min of 30 µM NMDA treatment (bottom panels). Arrows indicate examples of labeled mitochondria (left) or ER (right) that undergo shrinkage due to contraction or fission in response to NMDA. Scale bar represents 5 µm. (**C**,**D**) Same as in (**B**) but in the presence of FP801 (**C**) or FP802 (**D**). Arrowheads in D indicate examples of stable structures that remained largely unchanged after NMDA treatment. (**E**–**H**) Quantification of mitochondria (**E**,**F**) or ER (**G**,**H**) shape factor circularity and average organelle size before (white circles) and 2.5 min after (black circles) the addition of NMDA in the presence of FP801 or FP802. ***, **, ns represent *p*-values of <0.001, <0.01, not significant, respectively. Data are from three to four independent cell preparations.

We next investigated the morphological changes caused by NMDA bath application on hippocampal neurons using confocal live microscopy. Primary cultures were infected at DIV 8 with recombinant adeno-associated viruses containing expression cassettes for either mitochondrial-targeted mCherry or ER-targeted mNeonGreen (Figure 6A), and recorded on DIV 13. After recording 2 min of baseline, 30 µM NMDA was perfused for 2 min and then washed out. Between the end of the NMDA application and the first 30 s of washing, mitochondria changed from elongated to more compacted circular forms (Figure 6B, magenta, arrows on upper vs. lower panels), and the smooth-looking ER became bumpy with a bright dot-like appearance (Figure 6B, cyan, arrows on upper vs. lower panels). FP802, but not FP801, inhibited the effects of NMDA on mitochondria and ER networks, preserving the morphology observed during baseline recordings (Figure 6D, arrowheads). Measurements of mitochondrial and ER mean particle size and shape factor circularity were used to quantify the structural alteration induced by NMDA bath application and to illustrate the structure-protective activity of FP802 (Figure 6E–H).

### 3.4. NMDA-Induced Changes in ER–Mitochondria Contact Sites

**Figure 7:**
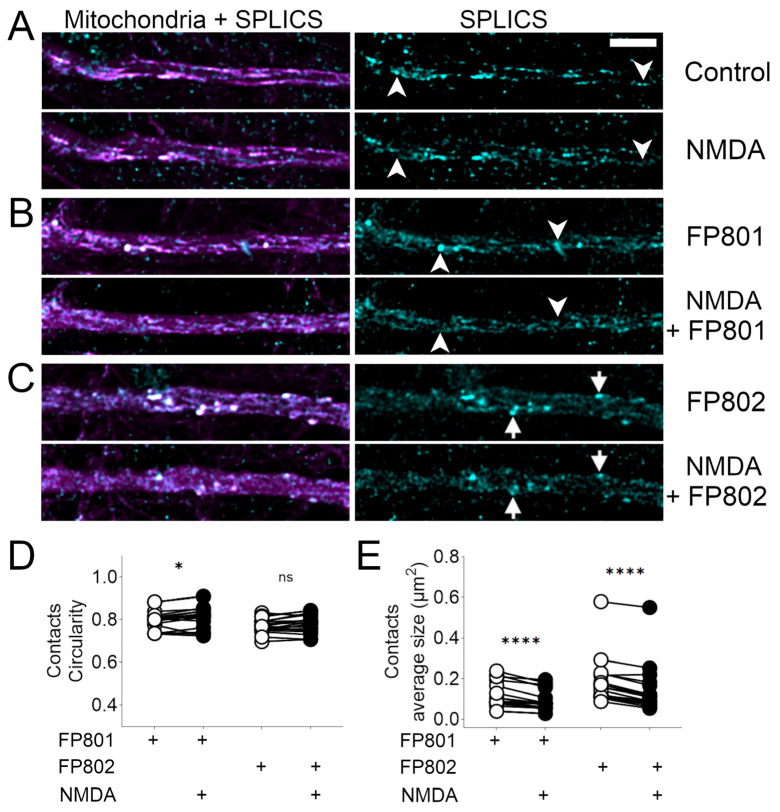
Partial loss of ER–mitochondria contact sites following NMDA bath application and inhibition by FP802. (**A**) Primary rat hippocampal neurons were infected on DIV 8 with rAAVs containing expression vectors for SPLICS (split-GFP-based contact site sensor) (cyan, right column) and the red mitochondrial marker (mito-mCherry) (magenta, left column). On DIV 13, neurons were transferred to an imaging chamber with constant perfusion and images were acquired every 2 s with a confocal microscope. The upper and lower panels show a dendrite before and after 2.5 min of 30 µM NMDA addition, respectively. (**B**,**C**) Same as in (**A**) but in the presence of 10 µM of FP801 (**B**) or 10 µM of FP802 (**C**). Arrowheads in (**A**,**B**) indicate examples of labeled ER–mitochondria contact sites that are lost after NMDA treatment. Arrows in (**C**) indicate examples of ER–mitochondria contact sites present before and, with reduced signal intensity, also after NMDA exposure in the presence of FP802. Scale bar, 5 µm. (**D**,**E**) Quantitative analysis of the circularity and average size of the contact sites before (white circles) and after NMDA treatment (black circles). ****, *, ns represent *p*-values of <0.0001, <0.05, not significant, respectively.

ER–mitochondria contact sites are signaling hubs that regulate mitochondrial function [15] and are involved in autophagy, apoptosis, and cancer [16,17], as well as neurodegenerative processes [18]. They can be detected in live imaging experiments using the recently developed split GFP-based indicators for detection of contact sites (SPLICS) [12]. These sensors rely on the reconstitution of a split variant of GFP, which fluoresces when its fragments are brought into close proximity. The fragments are fused to proteins that specifically localize to the membranes of two distinct organelles (e.g., mitochondria and the ER), enabling the detection of their interactions. We used rAAVs to express SPLICS and the mitochondrial marker mito-mCherry to identify dendritic ER–mitochondria contacts and followed their fate after NMDA exposure (Figure 7). We found that, in both control and FP801-treated neurons, ER–mitochondria contacts were readily detectable and largely resistant to NMDA challenge (Figure 7A,B). However, bright spots tended to disappear upon NMDA treatment (Figure 7A,B; arrowheads indicate bright spots sensitive to NMDA exposure). In the presence of FP802, the SPLICS signals appeared to be more stable and many bright spots even remained after NMDA treatment (Figure 7C, arrows indicate bright spots resistant to NMDA exposure). Measurements of circularity as a proxy for the shape of the contact sites indicated that FP802, but not FP801, prevented the NMDA-induced shape changes (Figure 7D). However, the SPLICS particle size became smaller after NMDA bath application, independently of the presence of FP802 (Figure 7E).

**Figure 8:**
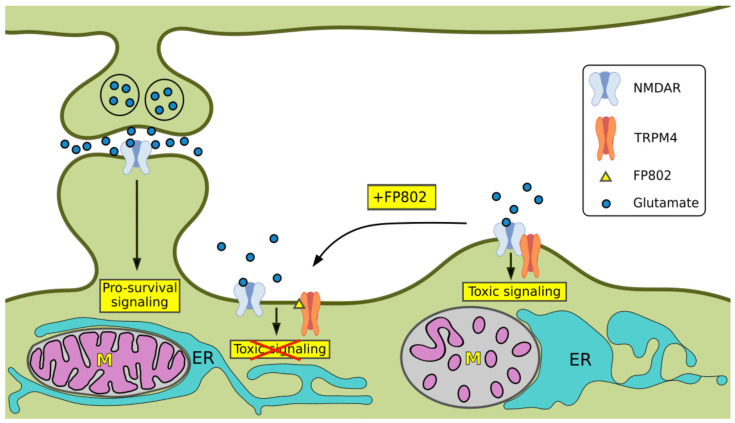
Schematic illustration of organelle damage associated with glutamate/NMDA neurotoxicity and its inhibition by the TwinF interface inhibitor FP802. Activation of toxic signaling by the extrasynaptic NMDAR/TRPM4 death signaling complex leads to structural alterations of mitochondria (indicated ‘M’) and ER, as well as partial loss of mitochondria–ER contact sites and dendritic blebbing. Organelle damage and dendritic blebbing associated with NMDAR/TRPM4-induced excitotoxicity is inhibited by FP802, which disrupts the NMDAR/TRPM4 complex, thereby detoxifying the extrasynaptic NMDARs. In contrast to extrasynaptic NMDARs, synaptic NMDARs promote pro-survival signaling, which involves stimulation of neuroprotective gene expression [2,19,20,21].

Our results support a model in which extrasynaptic NMDAR in complex with TRPM4 initiates glutamate/NMDA neurotoxicity-associated structural damage of dendrites and cell organelles (Figure 8). Disruption of the NMDAR/TRPM4 death complex using the TwinF interface inhibitor FP802 blocked dendritic blebbing, helped maintain the structural integrity of mitochondria and the ER, and mitigated the loss of ER–mitochondria contact sites.

## 4. Discussion

### 4.1. Organelle Damage Induced by the NMDAR/TRPM4 Complex

In this study, we established that activation of the extrasynaptic NMDAR/TRPM4 complex is primarily responsible for the dendritic blebbing and structural damage to mitochondria and the ER associated with NMDA-induced excitotoxicity. The damage includes in particular mitochondrial swelling and destruction of intramitochondrial architecture. The observed dramatic changes to mitochondrial cristae, which become swollen, disorganized, and heterogeneously distributed after NMDA challenge, are likely to be responsible for the mitochondrial dysfunction associated with glutamate/NMDA neurotoxicity [2,19,21]. Consistent with this causal relationship is the finding that NMDA treatment acting via the extrasynaptic NMDAR/TRPM4 complex leads to a rapid loss of the mitochondrial membrane potential [2,7]. Thus, destruction of mitochondrial cristae and loss of the mitochondrial membrane potential are among the earliest cell pathological events in glutamate/NMDA neurotoxicity. Despite the observed structural alteration of the ER upon NMDA challenge, ER–mitochondrial contact sites show significant resistance to disruption and remain at least partially intact during NMDA toxicity. Blebbing of dendrites appears to occur with slightly delayed kinetics compared to the organelle damage. Whether ER/mitochondrial damage and dendritic blebbing represent a sequentially occurring causal chain of events or whether the pathological changes occur independently of each other with different kinetics remains to be investigated.

### 4.2. Mechanism Underlying Toxic NMDAR/TRPM4 Signaling: “Two-Hit Hypothesis”

Our results indicate that the structural damage induced by the NMDAR/TRPM4 death complex likely involves a mechanism that extends beyond high intracellular calcium levels, emphasizing the need for a “two-hit hypothesis”. While calcium influx via extrasynaptic NMDARs is important for activating the damage cascade, one or several additional events induced by the NMDAR/TRPM4 death complex appear to be required to trigger the full spectrum of excitotoxicity-associated cell pathologies. Sodium flux through the NMDAR-associated TRPM4 channel may represent the ‘second signal’ that, together with calcium entering through NMDARs, initiates the damage, starting with deregulated mitochondrial ion homeostasis, loss of mitochondrial membrane potential, and changes in mitochondrial membrane fluidity compromising the electron transport chain and promoting superoxide anion formation, leading to osmotic imbalances and structural disintegration of cristae [22,23]. Disrupting the NMDAR/TRPM4 complex using the TwinF interface inhibitor FP802 may increase the physical distance between these two proteins, reducing the likelihood that calcium transients generated by activated extrasynaptic NMDARs can effectively activate TRPM4. TRPM4 activation requires very high calcium concentrations (EC_50_ 20.2 + 4 µM; measured using whole-cell recordings of HEK293 cells heterologously expressing TRPM4 [24]), which may only be reached when TRPM4 channels are in close proximity to NMDARs. By preventing the physical coupling of NMDAR and TRPM4, TwinF interface inhibitors may limit TRPM4 activation, thereby diminishing the sodium influx, which, in conjunction with the concomitant calcium increases, may be mitotoxic. This mechanism provides a possible explanation as to why FP802 prevents NMDA-induced mitochondrial dysfunction and promotes neuroprotection.

Also, in the context of dendritic blebbing, possible mechanisms and molecules in addition to calcium influx have been proposed. These include ionic imbalances, in particular dysregulated potassium and chloride homeostasis via pathways involving SK channels, K^+^-Cl^–^ cotransporter (KCC2) reversed uptake, or calcium-activated chloride channels [25,26]. Our finding that FP802 can prevent NMDA-induced dendritic blebbing indicates that this form of structural damage is also triggered by the NMDAR/TRPM4 death complex. It is conceivable that, similar to the damage to mitochondria, a high calcium/high sodium composite death signal triggers this cell pathology.

### 4.3. Organelle Damage in Human Neurodegenerative Diseases: TwinF Interface Inhibitors as Broad-Spectrum Neuroprotectants

This study shows striking parallels between organelle damage caused by the NMDAR/TRPM4 signaling complex and organelle damage observed in mouse models of neurodegenerative diseases and in post mortem brain tissue from patients with Alzheimer’s disease [27,28,29,30], ALS [31,32,33], Huntington’s disease [34,35,36], Parkinson’s disease [37,38], glaucoma [39], retinal degeneration [40], and stroke [41,42]. Notably, mitochondrial dysfunction and structural damage—a hallmark of toxic NMDAR/TRPM4 signaling—is common to virtually all neurodegenerative diseases and is believed to be a major promoter of disease progression [21,43]. It is possible that these pathological changes result from toxic NMDAR/TRPM4 signaling, as deregulation of glutamate uptake systems, and consequently elevated extracellular glutamate levels that stimulate extrasynaptic NMDARs, is common to many neurological disorders [44]. This raises the possibility that TwinF interface inhibitors may have therapeutic potential across a wide range of neurodegenerative conditions. Supporting this, a recent study demonstrated that FP802 can prevent the loss of spinal motor neurons in a mouse model of ALS [9]. Furthermore, FP802 reduced ALS-associated retinal ganglion cell degeneration and vision impairments [45] and may provide therapeutic benefits also in Alzheimer’s disease and Huntington’s disease, in which toxic NMDAR signaling has been implicated in disease progression [8,21,43]. Thus, TwinF interface inhibitors offer hope that currently untreatable human neurodegenerative diseases may become treatable.

## 5. Conclusions

Our findings highlight the critical role of the NMDAR/TRPM4 complex in mediating structural damage of dendrites and organelles in excitotoxicity. The requirement for a “two-hit” mechanism, which entails the involvement of both elevated calcium levels and a second signaling event to collectively instigate ionic and osmotic imbalances, underscores the complexity of this pathway. Disrupting the NMDAR/TRPM4 interaction with TwinF interface inhibitors presents a promising therapeutic strategy, potentially by attenuating the localized calcium signals near TRPM4 channels that are essential for their activation. These findings pave the way for targeted interventions to mitigate organelle damage and neuronal loss in a range of neurological disorders.

## 6. Patents

H.B. is the named inventor of the patent application for a novel class of neuroprotectants (PCT/EP2018/078577).

## Data Availability

The raw data supporting the conclusions of this article will be made available by the authors on request.
